# *Notes From the Field:* Campylobacteriosis Outbreak Associated with Consumption of Raw Water — Montana, 2022

**DOI:** 10.15585/mmwr.mm7215a6

**Published:** 2023-04-14

**Authors:** Rachel Hinnenkamp, Shawn Sorenson, Edward Evanson, Jonathan Yoder, Mia Mattioli

**Affiliations:** ^1^Public Health and Safety Division, Montana Department of Public Health and Human Services; ^2^Sanders County Environmental Health, Thompson Falls, Montana; ^3^Division of Foodborne, Waterborne, and Environmental Diseases, National Center for Emerging and Zoonotic Infectious Diseases, CDC.

Consumption of raw water (water that has not been disinfected or filtered) has become an emerging trend in the United States and could pose serious health consequences (*1*). Drinking water collected directly from outdoor freshwater sources such as lakes, rivers, and streams that has not been adequately treated (i.e., to remove pathogens) can cause disease and outbreaks (*2*). This report describes how a community in Western Montana responded to an outbreak of 19 cases of diarrheal illness associated with consuming untreated surface water.

On May 9, 2022, Sanders County, Montana, reported to the state health department six active cases of *Campylobacter* infection in their community; this case count represented a substantial increase above the 5-year average of six reported cases annually during 2017–2021. All infected persons reported drinking water from watering point A, an outlet of surface water from a creek near Paradise, Montana (Figure), before their onset of symptoms, which began on or after May 4. During the next 6 weeks, 13 additional cases of *Campylobacter jejuni* infection among persons exposed to the same water source were identified through laboratory testing (two by culture-independent confirmation and four by culture confirmation) or epidemiologic linkage (seven). One person was hospitalized, and no deaths were reported.

**FIGURE:**
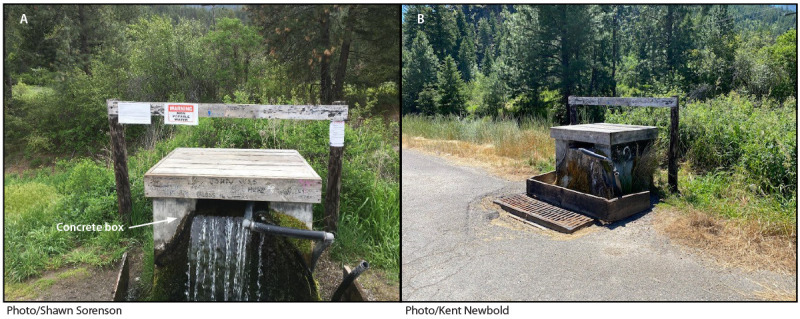
Watering point A, before any intervention (A) and after the water supply was permanently turned off (B) — Montana, 2022

On May 16, Sanders County Public Health environmental health staff members collected 23 liters of water from watering point A. The Montana Laboratory Services Bureau performed membrane filtration on 15 liters of the water sample, using four separate filters (0.45 *μ*m [44 mm] pore size). The filters were then plated on media for *Campylobacter* culture and isolation following standard methods (*3*); investigators did not culture for or find any other pathogens. On May 24, the water sample was confirmed positive for *Campylobacter* by culture. On June 3, staff members performed whole genome sequencing on one *Campylobacter* isolate from the water sample and isolates from two human outbreak specimens; sequences were compared by both core genome multilocus sequence typing and whole genome multilocus sequence typing (*4*). *Campylobacter* isolates from the human specimens and water samples were highly genetically related (0–1 allele apart). Together, whole genome sequencing analysis and epidemiologic data provided confirmatory evidence that this outbreak was the result of drinking water directly from watering point A.

Watering point A is located within the Montana Department of Transportation highway right-of-way on railroad property. The watering point was constructed, most likely during the early 1900s, to prevent the creek from eroding the track bed. Owners of adjacent land began using the water for domestic and agricultural purposes. Since then, the public has used watering point A as a drinking water source. Although watering point A contains untreated surface water, many community members believe that it is a natural spring. Users filled containers by placing them directly under water spilling out of the concrete box of the watering point, by placing containers directly into the water in the box, or by placing pumps or suction lines into the water to fill large containers. Signage posted by the Montana Department of Transportation before the outbreak warned the public that the watering point was not an approved public water source.

An unoccupied bird’s nest was found inside the box where the water sample was collected. Birds are a known source of *Campylobacter*, and although no birds were present at the time of sample collection, the presence of the nest indicates birds could have been the primary contamination source that led to this outbreak.

The combined strength of the epidemiologic, environmental, and laboratory evidence in this outbreak was sufficient to remove the watering source from operation. After a June 16 meeting with stakeholders, the Montana Department of Environmental Quality stated the source met the definition of a public water supply and therefore needed to meet the requirements of the Safe Drinking Water Act (*5*), or access had to be permanently removed. The Montana Department of Transportation permanently removed public access on June 28, 2022, by rerouting the creek water so that it remained underground (Figure). No additional cases have been identified since June 16, 2022. Persons drinking water from outdoor sources, including creeks, rivers, and streams, should always treat the water before drinking it. Boiling water is the most reliable way to kill germs, but treatment including filtration will also reduce the risk of illness from drinking water from outdoor sources.*

## Footnotes

*
https://www.cdc.gov/healthywater/drinking/travel/backcountry_water_treatment.html
